# Telomere length is maternally inherited and associated with lipid metabolism in Chinese population

**DOI:** 10.18632/aging.203810

**Published:** 2022-01-07

**Authors:** Liyun Guo, Yajuan Chen, Huiqin Li, Fanqian Yin, Mingxia Ge, Li Hu, Meiting Zi, Zhenghong Qin, Yonghan He

**Affiliations:** 1 Key Laboratory of Healthy Aging Research of Yunnan Province, Kunming Institute of Zoology, Chinese Academy of Sciences, Kunming 650201, China; 2 School of Rehabilitation, Kunming Medical University, Kunming 650500, China; 3 Department of Pharmacology and Laboratory of Aging and Nervous Diseases and Jiangsu Key Laboratory of Neuropsychiatric Diseases, College of Pharmaceutical Sciences, Soochow University, Suzhou 215123, China; 4 Kunming College of Life Science, University of Chinese Academy of Sciences, Beijing 100049, China; 5 College of Basic Medicine and Life Sciences, Hainan Medical University, Haikou 571199, China

**Keywords:** telomere length, heredity, visceral fat, lipid metabolism

## Abstract

Telomere is a unique DNA-protein complex which covers the ends of chromosomes to avoid end fusion and maintain the stability and integrity of chromosomes. Telomere length (TL) shortening has been linked to aging and various age-related diseases in humans. Here we recruited a total of 1031 Chinese individuals aged between 12 and 111 years, including 108 families with parents and their offspring. DNA was extracted from peripheral white blood cells and TL was measured by quantitative PCR (qPCR). We explored the associations of TL with age, gender and clinical variables, and tested the parental effects on TL variation. First, we found that TL was shortened with age, however, TL was better maintained in females than males. Second, there was a robust association of TL between mother and offspring, but not between father and their offspring. In addition, TL was inversely associated with visceral fat index in females, and positively associated with apolipoprotein A levels. Knockdown of the key genes for lipid metabolism (*PNPLA2* and *CPT1*) shortened the TL in HepG2 cells. These findings indicate that TL is maternally inherited, and impairment of lipid metabolism may contribute to the TL shortening in the Chinese population.

## INTRODUCTION

Telomere is a unique DNA-protein complex consisting of repetitive DNA sequences (TTAGGG_n_) and several associated protective proteins at the end of linear chromosomes [1, 2], which caps the chromosomal end and protects it from end-to-end fusing [3], consequently maintains DNA stability [4]. The telomere plays critical roles in meiotic chromosome segregation and chromosome silencing. Telomere length (TL) has been shown to associate with replicative potential. It was inversely associated with cell passage in cell culture, and with age at the organism level *in vivo* [3, 5, 6]. TL in blood cells has been studied extensively as a biomarker of human aging because its shortening is associated with lifespan [6, 7], and various age-related diseases [6, 8, 9]. Therefore, identifying determinants for TL has become a focus in telomere biology.

Factors influencing TL are not fully known, however it is likely that both genetic and various environmental factors, or their interactions play roles. For example, monozygotic and dizygotic twins studies indicate that mean TL and chromosome-specific TL patterns were in part inherited [10], with an estimated heritability ranging from 36% to 90% [11]. An earlier study reported that the inheritance of TL was associated with the X chromosome [12], but another two reports supported paternal inheritance of telomere [13, 14]. In contrast, there was a study reporting no significant differences in the father–offspring or mother–offspring TL correlation degree [15]. Thus far, the inheritance pattern of TL seems controversial and has been questioned.

Several other factors, such as obesity [16–18], pulse pressure [19], physical activity [20, 21], life stressors [22], and smoking [23], have been shown to impact TL, possibly through oxidative stress or inflammation [24, 25]. Likewise, the associations of these factors with TL are inconsistent across different studies. Most of the above studies were performed in Caucasians, and few of them were in East Asian population. Given the critical role of telomere in aging and age-related diseases, data from Asian populations are needed to identify and confirm the factors that may affect TL.

Here we explored the inheritance pattern of TL in 1031 Chinese individuals containing 108 families with parents and their offspring, and found a robust TL association between mothers and their offspring, supporting that TL is maternally inherited. Among the clinical variables, we showed that TL was inversely associated with visceral fat mass, but positively associated with apolipoprotein A (ApoA) levels, suggesting the TL is likely to be affected by lipid metabolism. Interference of the genes involved in lipid metabolism caused lipid accumulation and shortened TL *in vitro*. These findings suggest that TL is maternally inherited and is partially affected by lipid metabolism in Chinese.

## RESULTS

### General characteristics of the participants

Table 1 shows the basic characteristics of the studied population grouped by age at recruitment from -49 to 100+. Among them, 720 were females and 289 were males. Gender information in 5 subjects was unavailable. As shown in Table 1, most of the parameters were age-related (p<0.0001). Most of the indicators gradually increased or decreased along with age (Table 1), while some increased initially and then decreased with age, which is associated with the inclusion of the long-lived subjects (aged 90+) who usually have a specific metabolic profile as described in our and other studies [26–29]. Specifically, the levels of systolic blood pressure (SBP), alanine transaminase (AST) and creatinine (Cre) increased along with age; the opposite trend appeared in height, weight, bone mass (BOM), basic metabolism rate (BMR) and body fat rate (BFR). Surprisingly, the diastolic blood pressure (DBP), total cholesterol (TC), triglyceride (TG) and low-density lipoprotein (LDL) were improved at extreme age, which increased with age before 80 yr but decreased after 90 yr. In addition, high-density lipoprotein (HDL) and total bilirubin (TB) were not significantly changed among different age groups (Table 1).

**Table 1:** General clinical characteristics of the participants.

	**Age (y)**												
	**-49**		**50-59**		**60-69**		**70-79**		**90-99**		**100+**		***p*-value**
	N	Mean(±SD)	N	Mean(±SD)	N	Mean(±SD)	N	Mean(±SD)	N	Mean(±SD)	N	Mean(±SD)	
Height (cm)	30	154.8(±5.9)	53	150.6(±27.7)	77	153.1(±6.9)	35	153.7(±9.2)	187	144.1(±14.0)	12	143.8(±8.3)	0.000
Weight (kg)	30	57.2(±11.0)	53	57.2(±11.2)	75	54.1(±9.3)	35	53.5(±11.2)	188	40.6(±9.6)	12	41.0(±9.0)	0.000
Glu (mmol/L)	46	6.17(±1.69)	81	6.85(±2.41)	100	6.68(±1.72)	46	6.92(±1.87)	286	7.57(±2.48)	69	6.08(±1.29)	0.000
SBP (mmHg)	63	126(±20)	123	137(±21)	147	139(±20)	54	145(±22)	366	144(±25)	109	145(±22)	0.000
DBP (mmHg)	63	83(±13)	123	89(±13)	147	88(±13)	54	91(±15)	366	82(±15)	109	81(±14)	0.000
TC (mmol/L)	67	5.04(±1.47)	125	5.47(±1.74)	149	5.69(±2.19)	49	5.98(±1.23)	417	5.05(±1.30)	108	4.71(±1.57)	0.000
TG (mmol/L)	73	1.77(±1.16)	135	2.38(±2.00)	166	2.15(±1.50)	51	2.16(±2.02)	417	1.61(±1.08)	108	1.64(±1.07)	0.000
HDL (mmol/L)	73	1.49(±0.40)	136	1.53(±0.40)	168	1.68(±1.49)	51	1.42(±0.35)	417	1.47(±0.36)	108	1.50(±0.47)	0.08
LDL (mmol/L)	73	3.22(±0.93)	136	3.54(±1.11)	167	3.44(±1.01)	53	3.84(±0.94)	416	3.21(±0.91)	108	2.86(±0.97)	0.000
ALT (U/L)	40	21.4(±13.6)	75	24.7(±20.9)	114	22.0(±11.3)	46	23.5(±13.0)	256	12.2(±8.4)	68	20.5(±8.4)	0.000
AST (U/L)	40	24.8(±9.41)	76	29.6(±13.9)	111	28.5(±8.92)	45	29.0(±10.0)	254	25.8(±9.45)	68	32.3(±10.6)	0.000
ALP (U/L)	30	60.9(±45.9)	52	62.2(±24.4)	75	74.8(±23.0)	33	73.0(±20.6)	193	71.9(±31.5)	13	78.3(±22.2)	0.083
TB (μmol/L)	40	10.2(±5.9)	76	11.0(±4.4)	114	10.8(±4.9)	46	10.6(±4.3)	258	11.1(±4.2)	69	9.4(±4.6)	0.222
Cre (μmol/L)	40	59.6(±14.0)	76	69.2(±19.5)	115	72.6(±19.4)	47	77.7(±19.2)	258	83.3(±26.1)	69	90.0(±26.3)	0.000
UA (μmol/L)	33	304(±83)	65	322(±93)	95	347(±92)	44	362(±90)	197	362(±94)	69	330(±86)	0.000
BMI (kg/m^2^)	30	23.8(±3.9)	53	23.9(±3.6)	76	23.0(±3.4)	35	22.2(±3.5)	187	19.2(±3.6)	12	19.7(±3.5)	0.000
BMR (kJm^-2^h^-1^)	29	1160(±138)	53	1168(±242)	76	1112(±191)	35	1099(±223)	187	835(±182)	12	864(±146)	0.000
BFR (%)	30	29.9(±8.9)	52	28.0(±9.6)	76	27.8(±8.4)	35	26.1(±7.1)	187	24.8(±9.4)	12	22.4(±10.6)	0.006
PBW (%)	29	51.4(±5.9)	53	52.5(±5.5)	76	51.8(±4.8)	35	51.7(±6.3)	187	48.1(±2.9)	12	54.2(±10.4)	0.000
VFI (kg/m^2^)	29	5.28(±3.00)	52	7.07(±3.54)	76	7.30(±3.40)	34	8.39(±4.37)	187	8.53(±3.81)	12	6.96(±2.64)	0.000
BOM (kg)	30	2.26(±0.31)	53	2.32(±0.37)	76	2.11(±0.38)	35	2.11(±0.51)	187	1.45(±0.49)	12	1.56(±0.44)	0.000

Abbreviations: Glu, blood glucose; SBP, systolic blood pressure; DBP, diastolic blood pressure; TC, total cholesterol; TG, triglyceride; HDL, high-density lipoprotein; LDL, low-density lipoprotein; ALT, alanine transaminase; AST, aspartate transaminase; ALP, alkaline phosphatase; TB, total bilirubin; Cre, creatinine; UA, uric acid; BMI, body mass index; BMR, basic metabolism rate; BFR, body fat rate; PBW, percent body water; VFI, visceral fat index; BOM, bone mass.

### The telomere length is better maintained in females than males

**Figure 1:**
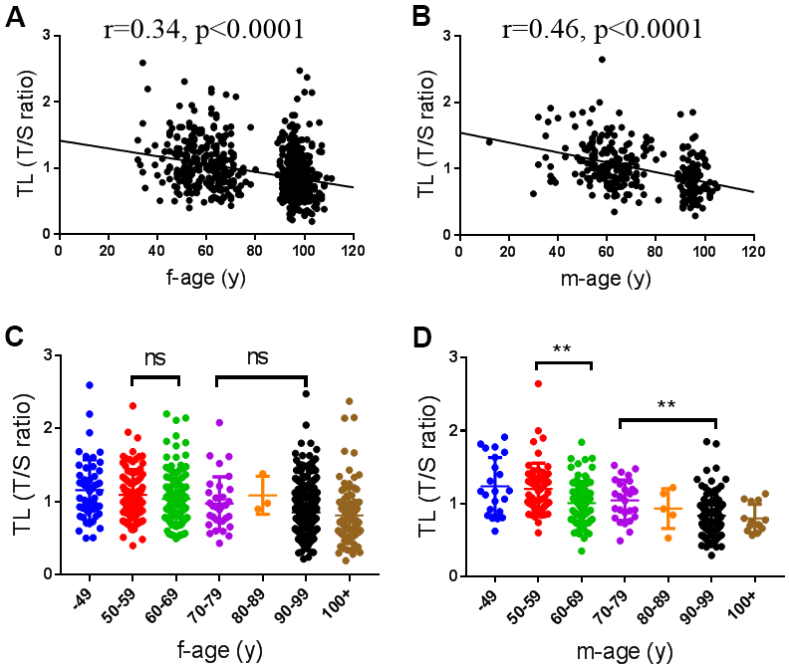
**Associations of telomere length (TL) with age.** (**A**, **B**) Scatter plot of TL with age in female (n=720) and male subjects (n=289), respectively. (**C**, **D**) The distribution of TL in female and male at different age groups. f, female; m, male.

Since the TL is a marker of aging, we first analyzed the changes of TL with age from 49 to 100+ yr. As shown in Figure 1, TL was negatively correlated with age in both male and female subjects as expected (female: r=0.34, p<0.0001, Figure 1A; male: r=0.46, p<0.0001, Figure 1B), but the correlation between TL and age was significantly higher in males than in females (p=0.038). There were not any differences in TL between neighboring age groups before 90 years in females (Figure 1C). In males, TL was significantly shortened in 60-69 and 90-99 age groups than 50-59 and 70-79 age groups, respectively (Figure 1D). However, there were not significant gender differences in the mean TL among different age groups (Supplementary Table 1). These observations suggest that telomere attrition may be accelerated in males than females when they are old.

### TL is maternally inherited

TL has been reported to be affected by genetic factors in Caucasian populations [10, 12, 13]. Here we tested the association of TL between parents and their F1 offspring to see whether there is a genetic basis for TL. As shown in Table 2 and Supplementary Figure 1, there was a significant correlation in TL between mother and offspring (r=0.44, p=0.0001, Supplementary Figure 1A and Table 2). Among them, the mother-daughter TL correlation was higher than mother-son TL correlation (mother-daughter: r=0.62, p=0.01, Supplementary Figure 1B and Table 2; mother-son: r=0.35, p=0.01, Supplementary Figure 1C and Table 2). However, we did not observe any correlations between father and their offspring (r=0.23, p=0.36, Supplementary Figure 1D and Table 2), neither between father–daughter (r=0.41, p=0.36, Supplementary Figure 1E and Table 2) nor father–son (r=0.19, p=0.46, Supplementary Figure 1F and Table 2). These findings suggest that TL is maternally inherited in the Chinese population.

**Table 2:** Age-adjusted intrafamilial correlations of telomere length (TL).

**Relationship**	**No. of pairs**	***r***	***p*-value**
**Mother-children**	76	0.44	0.0001
Mother-daughter	19	0.62	0.01
Mother-son	57	0.35	0.01
**Father-children**	32	0.23	0.36
Father-daughter	10	0.41	0.36
Father-son	22	0.19	0.46

### Telomere length is related to lipid metabolism

**Figure 2:**
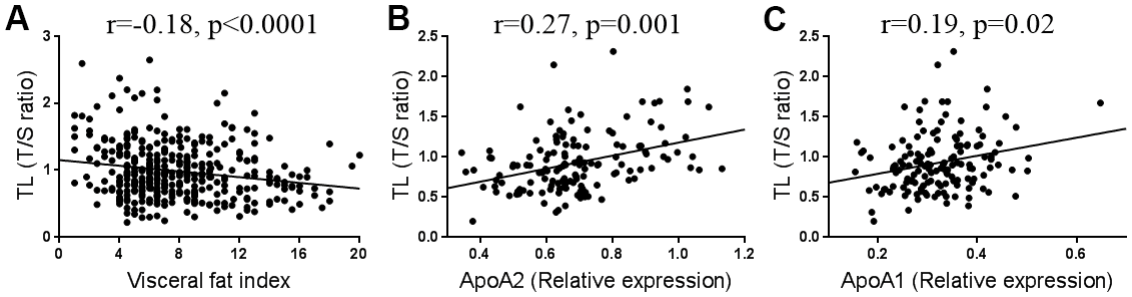
Correlation between telomere length (TL) and visceral fat (n=394) (**A**), ApoA2 (n=144) (**B**), and ApoA1 levels (n=144) (**C**).

**Figure 3:**
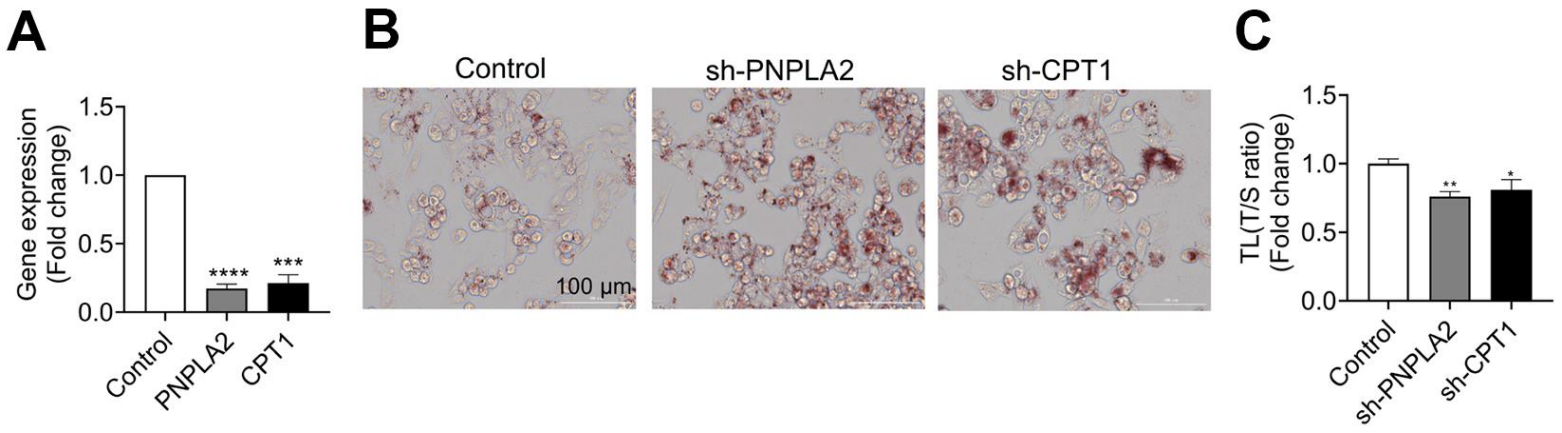
**Knockdown of genes for lipid metabolism shortened telomere length (TL) in HepG2 cells.** *PNPLA2* and *CPT1* genes were knocked down by short-hairpin RNA (shRNA) in HepG2 cells. (**A**) Efficiency of gene knockdown by short-hairpin RNA (shRNA) was evaluated by qPCR. (**B**) Oil red O staining in HepG2 cells after *PNPLA2* and *CPT1* knockdown by shRNA. (**C**) TL was determined by qPCR in HepG2 cells. Data were expression as mean±SEM. *p<0.05, **p<0.01, ***p<0.001, ****p<0.0001.

In order to identify potential factors that may affect TL, we tested the correlations between TL and various clinical indicators in Table 1. As shown in Table 3, most clinical variables, such as blood glucose (Glu), blood pressure, or blood lipids were not statistically associated with TL, except for percent body water (PBW, %) (r=0.15, p=0.004) and visceral fat index (VFI) (r=-0.18, p<0.0001). Interestingly, the correlations existed only in females but not in males (Table 3). In addition, we found that AST, an index that clinically reflects the liver function, was weakly but significantly correlated with TL in females (r=-0.10, p=0.043, Table 3). Among the TL-associated factors, VFI has the strongest correlation with TL (Table 3 and Figure 2A). Furthermore, we found that genes related to lipid metabolism were highly co-expressed with those related to TL maintenance (Supplementary Table 2). As liver is the major organ for lipid metabolism, we infer that TL is likely to be affected by lipid metabolism. To test this, we randomly selected 144 samples to measure their serum proteins by proteomics. After adjustment for age, the most relevant proteins with TL were apolipoproteins A (ApoA), including ApoA2 (r=0.27, p=0.001, Figure 2B) and ApoA1 (r=0.19, p=0.02, Figure 2C). As ApoA are critically involved in lipid metabolism in the liver [30], we tested the TL after interfering the key genes (*PNPLA2* and *CPT1*) in a liver-derived cell line HepG2 (Figure 3A), and found that knockdown of either genes sufficiently caused lipid accumulation (Figure 3B), and shortened the TL (Figure 3B, 3C). These findings suggest that impairment of lipid metabolism affects the TL in the liver.

**Table 3:** Correlations between telomere length and indicators involved.

	**r(all)**	***p*-value**	**r(f)**	***P*-value**	**r(m*)***	***p*-value**
height(kg)	0.02	0.71	0.04	0.51	-0.02	0.885
BMI(kg/m^2^)	-0.08	0.096	-0.07	0.222	-0.12	0.280
BM kJ/(m2·h)	-0.06	0.23	-0.08	0.188	-0.01	0.902
BFR(%)	-0.09	0.09	-0.07	0.241	-0.16	0.137
**PBW(%)**	0.15	0.004	0.14	**0.018**	0.17	0.110
**VFI**(kg/m^2^)	-0.18	0.000	-0.21	**0.000**	-0.10	0.350
BOM(kg)	-0.02	0.63	-0.04	0.530	0.06	0.613
weight(kg)	-0.08	0.14	-0.08	0.184	-0.05	0.658
Glu(mmol/L)	0.01	0.77	-0.01	0.789	0.13	0.119
SBP(mmhg)	-0.02	0.66	-0.03	0.474	0.03	0.699
DBP(mmhg)	-0.03	0.45	-0.03	0.437	-0.01	0.922
TC(mmol/l)	0.03	0.33	0.04	0.294	-0.01	0.932
TG(mmol/L)	0.001	0.99	-0.02	0.658	0.03	0.591
HDL(mmol/L)	-0.04	0.22	-0.03	0.455	-0.12	0.051
LDL(mmol/L)	0.05	0.16	0.07	0.06	-0.04	0.558
ALT(U/L)	-0.05	0.27	-0.07	0.118	0.03	0.753
**AST(U/L)**	-0.08	0.07	-0.10	**0.043**	-0.02	0.770
ALP(U/L)	0.04	0.411	0.06	0.287	-0.04	0.694
TB(μmol/L)	0.07	0.098	0.07	0.123	0.05	0.575
Cre(μmol/L)	-0.03	0.47	-0.03	0.597	-0.06	0.476
UA(μmol/L)	-0.03	0.56	-0.04	0.46	-0.003	0.972

Parameters and values in bold represent statistically significant correlation.

Abbreviations: all, all samples; f, female; m, male; BMI, body mass index; BMR, basic metabolism rate; BFR, body fat rate; PBW, percent body water; VFI, visceral fat index; BOM, bone mass; Glu, blood glucose; SBP, systolic blood pressure; DBP, diastolic blood pressure; TC, total cholesterol; TG, triglyceride; HDL, high-density lipoprotein; LDL, low-density lipoprotein; ALT, alanine transaminase; AST, aspartate transaminase; ALP, alkaline phosphatase; TB, total bilirubin; Cre, creatinine; UA, uric acid.

## DISCUSSION

Although the general characteristics and influencing factors of TL have been reported in multiple Caucasian populations, data are still lacking in Chinese population. In this study, we analyzed the relationship of TL with age, gender, and the potential influencing factors, as well as the inheritance pattern of TL across generations in a Chinese population for the first time. We found that TL decreased along with age, but was better maintained in females than males. Moreover, we showed that TL was maternally inherited, with a robust relation between mother and their offspring. We further revealed that TL is related to lipid metabolism, such as visceral fat accumulation, liver function and serum ApoA protein levels. Thus, telomere maintenance is likely determined by genetic factors and shaped by non-genetic influences throughout human life.

Some studies have found that females have longer TL than males [12, 31, 32], which is likely associated with the protective role of oestrogen in stimulating telomerase to add telomere repeats to the ends of chromosomes [12]. In addition, telomeres are very sensitive to oxidative stress [33], and women produce fewer reactive oxygen species (ROS) than men [12]. However, it is not always the case that TL is longer in women than men [34, 35] or even the reverse [36]. In our study, although there were not any differences in TL between men and women in the same age group, we found that TL was better maintained in females than males with the age increase. TL was dynamically attritted with age and affected by a variety of environmental factors, such as occupational exposures [37], household income [35], and lifestyle (dietary patterns, drinking, smoking, and physical activity) [38–40]. Generally, males have higher risk to occupational exposures, higher rate of drinking and smoking, accounting for their accelerated telomere attrition in relative to females.

The inheritance pattern of telomere has been reported in several studies. A cross-sectional study and meta-analysis involving 19,713 European subjects aged 15-99 years showed that maternal inheritance of TL is stronger than paternal inheritance [41]. Based on the whole-genome sequencing of the Dutch population, Nersisyan et al. showed that TL tends to be inherited maternally [42]. A previous study based on a population of northern Belgium reported that the inheritance of TL was linked to the X chromosome [12]. However, several studies showed that TL was inherited from the paternal line or no parental bias [13, 14, 43]. These discrepancies may be attributed to differences in the genetic background or health status of the recruited subjects. Our results support that TL is maternally inherited in the Chinese population, which is the first study to test the inheritance pattern of TL in Chinese. We found a significant inheritance correlation between mother and their offspring, either in mother-daughter or mother-son comparisons. Interestingly, the mother-daughter TL correlation was higher than mother-son TL correlation. The mechanisms have yet to be investigated, but the beneficial effect of oestrogen on telomere maintenance [12] and lower occupational exposures in females [44], as well as the XX sex chromosome effect [45] may act in the differential correlation. In contrast, we did not observe any correlations for TL between father and their offspring. Due to the Chinese traditional culture, parents usually live with their sons when their children grow up and get married. Though the sample number of father-daughter pairs was too small to test the heritability between father and daughter, neither the correlation was there between father-son TL even the sample number was acceptable. Totally, our study suggests that TL is inheritable in Chinese population from the maternally line.

Many other influencing factors for TL have been reported, including cardiovascular diseases, diabetes and obesity, etc. [40, 46–48]. Here we found that TL is negatively correlated with visceral fat, and positively correlated with some ApoA levels even after age adjustment. Some studies have reported an inverse relationship between body mass index (BMI) and TL [16, 18], which was not observed in our study. Obesity defined by BMI is remarkably heterogeneous. People with similar body weight or BMI values can have substantially different comorbidities and levels of health risk [49]. Instead, visceral fat mass may be the real culprit that links telomeres with various metabolic diseases. Data from several epidemiological studies have shown that visceral adipose tissue is an independent risk factor of morbidity and mortality [49]. Emerging evidence also suggest that visceral fat deposition contributes to increased atherosclerosis and cardiometabolic risk [50]. Moreover, obesity is a key factor in the development of metabolic abnormalities [51], including metabolic syndrome, systemic chronic inflammation and oxidative stress. Apolipoproteins were closely related to lipid metabolism and affect body fat distribution [52]. The ApoA-TL correlation lends additional support to that TL is likely to be affected by lipid metabolism. Additionally, we found that AST, an indicator of liver function, was inversely related with TL. Consistent with previous studies [16], we found the correlation between TL and lipid metabolism existed only in females, which is possibly associated with sex hormone or other yet unknown X chromosome-harboring genes. There is an oestrogen-responsive element in human telomerase reverse transcriptase (hTERT) [53], so the hormone may stimulate telomerase and further maintain TL in females. In addition, the X chromosome harbors some genes, such as the DKC1 and AGTR2, which are important for maintaining the telomerase activity [54, 55]. TL is likely to affect female health by mutually interacting with lipid metabolism. As telomere attrition is implicated in cellular senescence, TL maintenance may facilitate the homeostasis of lipid metabolism via regulating cellular senescence in adipose tissue [56]. On the other hand, excess adipose tissue can induce chronic and systemic inflammatory state [57], which can cause TL shortening by inducing ROS production and resultant oxidative stress [24]. However, the casual relationship between TL and lipid metabolism has yet to be investigated. Our preliminary data revealed that knockdown of the key genes for lipid metabolism caused fat accumulation and significantly shortened the TL in the liver cells, lending additional support to the observations in the population study. Nevertheless, more mechanistic studies are needed to explain the gender-specific differences and influencing factors for TL in the future.

Above all, our findings indicate that TL is maternally inherited, and impairment of lipid metabolism may contribute to the TL shortening in the Chinese population.

## MATERIALS AND METHODS

### Study population

In this study, 1031 participants aged from 12 to 111 years from Hainan Province (785 subjects) and Hubei Province (246 subjects) of China were recruited. The participants included 547 healthy elderly individuals, 188 offspring of the old (F1), 236 spouses of F1 (F1SP). The familial information was not available for the rest 60 individuals, who were not included into the inheritance analysis. A total of 108 families were used to assess the association of TL between the parents and their F1 offspring. Identity card was used to verify the participants’ age and written informed consent was obtained from each of the participants. The study protocol was approved by the Ethics Committee at Kunming Institute of Zoology, Chinese Academy of Sciences.

### Telomere length measurement

We collected blood samples from all participants and stored them in a −30° C refrigerator. Genomic DNA was extracted from blood samples by AxyPrep Blood Genomic DNA Miniprep Kit (Corning, NY, USA) and stored at −80° C. We measured the average TL by quantitative PCR (qPCR) as described by Cawthon et al. [58]. Briefly, the standard curves of TL and single gene (β-globin gene, HGB) amplification response were obtained from a reference DNA sample that continuously diluted with double distilled water by 4-fold per dilution to obtain 4 concentrations of DNA ranging from 0.94 to 60 ng/μL. Reference DNA comprised mixed DNA from 60 individuals who were randomly selected from the 1031 subjects in this study. Each PCR reactions were carried out using about 20 ng DNA in a 20 μL volume by FastStart Essential DNA Green Master (Roche, Basle, Switzerland). The primer sequences are TELF, 5’-CGGTTTGTTTGGGTTTGGGTTTGGGTTTGGGTTTGGGTT-3’; TELR, 5’-G GCTTGCCTTACCCTTACCCTTACCCTTACCCTTACCCT-3’; HGBF, 5’-GCTT CTGACACAACTGTGTTCACTAGC-3’; and HGBR, 5’-CACCAACTTCATCCAC GTTCACC -3’. Cycling conditions were 95° C for 10 min followed by 30 cycles of 95° C for 10 sec and 56° C for 30 sec for TEL, 95° C for 10 min followed by 35 cycles of 95° C for 10 sec and 58° C for 20 sec for HGB. TL was expressed as T/S where T indicates telomere repeats (T) and S indicates the copy number of single copy genes (S). T/S values were calculated by the formula 2^-ΔΔCt^, where ΔΔCt=C_t (samples)_^telomere^ - C_t (samples)_^β-globin^ - C_t (reference)_^telomere^ - C_t (reference)_^β-globin^.

### Biochemical measurements of peripheral blood

Blood biochemical indicators were tested in clinical laboratories of local hospitals. 2 mL peripheral blood sample for each subject was sent to hospital within three hours after collecting. The indicators included blood glucose (Glu), total cholesterol (TC), triglyceride (TG), high-density lipoprotein (HDL), low-density lipoprotein (LDL), alanine transaminase (ALT), aspartate transaminase (AST), alkaline phosphatase (ALP), total bilirubin (TB), creatinine (Cre), and uric acid (UA).

### Measurements of body composition

Body composition was measured by a body fat scale (InBody, Cheonan city, South Korea). The working principle of the scale was described previously [59]. Briefly, subjects were required to stretch out their arms to avoid contact with waist, put their thumbs on the round electrodes, place their heels at the end of the heel electrodes. Body compositions included body mass index (BMI), basic metabolism rate (BMR), body fat rate (BFR), percent body water (PBW), visceral fat index (VFI), and bone mass (BOM).

### Measurements of serum proteins

Relative proteins were quantified using Tandem Mass Tag mass spectrometry by PTM BIOLABS company. Firstly, the top 12 high abundance proteins were removed from serum samples by Pierce Top 12 Abundant Protein Depletion Spin Columns Kit (Thermo Fisher Scientific, Waltham, MA, USA). Trypsin was then used for the protein digestion. After trypsin digestion, peptide was desalted by Strata X C18 SPE column (Phenomenex, CA, USA) and labelled according to the manufacturer’s protocol for TMT kit/iTRAQ kit (Thermo Fisher Scientific, Waltham, MA, USA). Then, the tryptic peptides were fractionated into fractions by high pH reverse-phase HPLC using Thermo Betasil C18 column. The peptides were subjected to NSI source followed by tandem mass spectrometry (MS/MS) in Q ExactiveTM Plus (Thermo Fisher Scientific, Waltham, MA, USA) coupled online to the UPLC. Finally, the resulting MS/MS data were processed using Maxquant search engine (v.1.5.2.8). Tandem mass spectra were searched against the Uniprot database concatenated with reverse decoy database.

### Cell culture, short-hairpin RNA (shRNA), Oil Red O staining and measurement of TL

HepG2 and HEK-293T cells were cultured in Dulbecco’s modified Eagle medium (Sigma-Aldrich, St. Louis, MO, USA) supplemented with 10% heat-inactivated fetal bovine serum (Thermo Fisher Scientific, Waltham, MA, USA), and 1% penicillin/streptomycin (Thermo Fisher Scientific, Waltham, MA, USA) in a humidified incubator at 37° C and 5% CO_2_. shRNAs targeting PNPLA2 and CPT1 were cloned into EGFP-pLKO.1 lentiviral vector. The lentiviruses were harvested from HEK-293T cells, and then transfected into HepG2 cells. Infected cells were selected for 3 passages in medium containing 2 μg/mL puromycin. The efficiency of shRNA knockdown was evaluated by qPCR. Sequences used for shRNA and primers for qPCR were listed in Supplementary Table 3. After knockdown of the target genes, lipid was stained with Oil Red O (Sigma-Aldrich, St. Louis, MO, USA) and cell photos were taken using Nikon inverted microscope. TL was measured using qPCR after PNPLA2 and CPT1 knockdown in HepG2 cells according to the method as described [60].

### Data analysis

Most of the statistical analyses were performed with SPSS 21.0 (SPSS Inc., Chicago, IL, USA). Continuous variables were expressed as mean±SD and compared by the One-Way ANOVA or nonparametric test according to the distribution of data. Multiple linear regression was used to evaluate the contribution of multiple indicators to TL. The correlation coefficient between TL and indicators involved was assessed by Pearson correlation coefficient or partial correlation analysis (adjusted age and gender). Significance of the difference between the age-TL correlation coefficients in females and males were tested using an online tool (http://vassarstats.net/rdiff.html). The expression data for genes involved in lipid metabolism and TL maintenance were extracted from the RNA-seq data (not shown) in our lab, and the associations between two gene sets were measured by Pearson correlation analysis in R platform. Data of gene knockdown and TL in cell experiments were expressed as mean±SEM and analyzed using the GraphPad Prism 7 software (GraphPad Software, La Jolla, CA, USA). All comparisons were made by two-tailed. *P* values less than 0.05 were considered statistically significant.

## Supplementary Material

Supplementary Figure 1

Supplementary Table 1

Supplementary Table 2

Supplementary Table 3
