# Supplementary material for: Telomere length is maternally inherited and associated with lipid metabolism in Chinese population
Source: Aging (Albany NY). 2022 Jan 7;14(1):354–67. doi: 10.18632/aging.203810 (PMC8791204; doi:10.18632/aging.203810)
Supplement: Supplementary Figure 1 [file aging-14-203810-s001.pdf]

SUPPLEMENTARY FIGURE

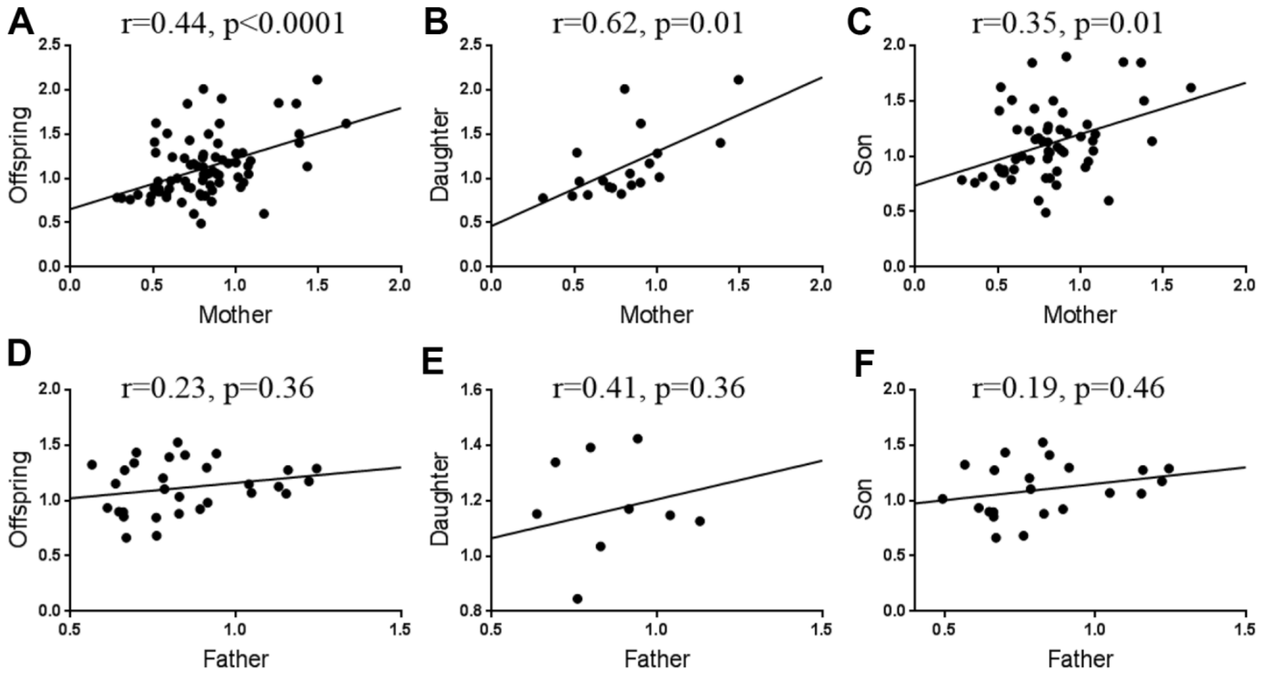

Supplementary Figure 1. Intrafamilial associations of telomere length. (**A**) mother vs. offspring. (**B**) mother vs. daughter. (**C**) mother vs. son. (**D**) father vs. offspring. (**E**) father vs. daughter. (**F**) father vs. son.
