# Supplementary material for: Telomere length is maternally inherited and associated with lipid metabolism in Chinese population
Source: Aging (Albany NY). 2022 Jan 7;14(1):354–67. doi: 10.18632/aging.203810 (PMC8791204; doi:10.18632/aging.203810)
Supplement: Supplementary Table 1 [file aging-14-203810-s002.pdf]

Supplementary Table 1. Telomere length distribution by gender and age.

Age (y)	Tel-all		Tel-female		Tel-male	
	N	mean(±SD)	N	mean(±SD)	N	mean(±SD)
-49	74	1.19±0.40	52	1.17±0.41	22	1.24±0.39
50-59	149	1.15±0.34	95	1.11±0.33	54	1.21±0.35
60-69	177	1.04±0.34	105	1.05±0.38	72	1.03±0.28
70-79	58	1.01±0.33	32	0.98±0.37	26	1.04±0.28
80-89	8	0.99±0.26	3	1.09±0.26	5	0.93±0.27
90-99	428	0.85±0.31	331	0.85±0.31	97	0.83±0.29
100+	115	0.81±0.37	102	0.81±0.38	13	0.76±0.18
total	1009	0.96±0.36	720	0.94±0.37	289	1.00±0.34

Note: Among the 1031 participants, 22 did not have either age or gender information so that they were not included for the analysis in the table.
