# Supplementary material for: Telomere length is maternally inherited and associated with lipid metabolism in Chinese population
Source: Aging (Albany NY). 2022 Jan 7;14(1):354–67. doi: 10.18632/aging.203810 (PMC8791204; doi:10.18632/aging.203810)
Supplement: Supplementary Table 3 [file aging-14-203810-s004.pdf]

**Supplementary Table 3. Sequences used for shRNA and primers for qPCR in the *in-vitro* study.**

| | Forward | Reverse |
|---|---|---|
| shPNPLA2 | CCGGGCCAAGTTCATTGAGGTATCTCTCG AGAGATACCTCAATGAACTTGGC TTTTTG | AATTCAAAAAGCCAAGTTCATTGAGGTATC TCTCGAGAGATACCTCAATGAACTTGGC |
| shCPT1 | CCGGCGATGTTACGACAGGTGGTTTCTCG AGAAACCACCTGTCGTAACATCG TTTTTG | AATTCAAAAACGATGTTACGACAGGTGGTT TCTCGAGAAACCACCTGTCGTAACATCG |
| qPCR-PNPLA2 | GAGATGTGCAAGCAGGGATAC | CTGCGAGTAATCCTCCGCT |
| qPCR-CPT1 | ATCAATCGGACTCTGGAAACGG | TCAGGGAGTAGCGCATGGT |
